# Paraneoplastic Pemphigus Involving the Respiratory and Gastrointestinal Mucosae

**DOI:** 10.1155/2020/7350759

**Published:** 2020-06-17

**Authors:** Kentaro Odani, Akane Itoh, Soshi Yanagita, Yasuhito Kaneko, Mitsuhiro Tachibana, Takashi Hashimoto, Yutaka Tsutsumi

**Affiliations:** ^1^Department of General Medicine, Shimada Municipal Hospital, Shimada, Shizuoka, Japan; ^2^Department of Diagnostic Pathology, Shimada Municipal Hospital, Shimada, Shizuoka, Japan; ^3^Department of Diagnostic Pathology, Kyoto University Hospital, Kyoto, Japan; ^4^Department of Pediatrics, Hamamatsu University School of Medicine, Hamamatsu, Shizuoka, Japan; ^5^Department of Hematology, Shimada Municipal Hospital, Shimada, Shizuoka, Japan; ^6^Department of Dermatology, Shimada Municipal Hospital, Shimada, Shizuoka, Japan; ^7^Department of Dermatology, Osaka City University Graduate School of Medicine, Osaka, Japan; ^8^Diagnostic Pathology Clinic, Pathos Tsutsumi, Nagoya, Aichi, Japan

## Abstract

Paraneoplastic pemphigus (PNP), an autoimmune mucocutaneous disorder involving the oral and bronchial mucosae, is a rare complication of hematologic malignancy. Serologically, serum autoantibodies against varied desmosome-related proteins are of notice. PNP is often lethal due to bronchiolitis obliterans and opportunistic infection. A 70-year-old Japanese male complained of dry cough, stomatitis, and sore throat. The lips and oral mucosa were severely eroded, and skin eruptions were seen on the chest and abdomen. The biopsy features were consistent with PNP, and the deposition of IgG and IgM was shown on the plasma membrane of the involved keratinocytes. Serological studies demonstrated autoantibodies to desmoglein-3, desmocollins-2 and -3, bullous pemphigoid antigen-1, envoplakin and periplakin. Systemic evaluation disclosed mantle cell lymphoma, stage 4B. After chemotherapy, partial remission was reached. PNP was treated with methylprednisolone and intravenous immunoglobulins, and the oral lesion only temporarily responded. He died of respiratory failure two months after onset. Autopsy revealed residual indolent lymphoma and systemic opportunistic infections. *Aspergillus* colonized the eroded bronchial/bronchiolar mucosa, associated with extensive vascular invasion. Coinfection of cytomegalovirus (CMV) and *Pneumocystis jirovecii* caused interstitial pneumonia. The oropharyngeal, respiratory, esophageal, and gastrointestinal mucosae were diffusely infected by CMV. Bronchiolitis obliterans was observed in the peripheral lung. PNP-related acantholysis-like lesions were microscopically identified in the bronchial and gastrointestinal mucosa. IgG deposition and cleaved caspase-3-immunoreactive apoptotic cell death were proven in the involved mucosal columnar cells. Pathogenesis of the mucosal involvement is discussed.

## 1. Introduction

Paraneoplastic pemphigus (PNP), an autoimmune mucocutaneous disorder, is a rare complication of malignancy, first described by Anhalt et al. [1]. The disease commonly occurs in patients aged between 45 and 70 years, and the male to female ratio is around 1 : 1 [2]. PNP accompanies a poor prognosis: the mortality rate reaches 90% [2] and most of the patients die within a year after diagnosis, often with immunosuppression-related opportunistic infection [3].

PNP is commonly associated with hematologic neoplasms: non-Hodgkin's lymphoma (39%), chronic lymphocyte leukemia (18%), Castleman's disease (18%), carcinoma (9%), thymoma (6%), sarcoma (6%), and others (4%) [4]. In one-third of cases, PNP manifests mucocutaneous lesions prior to tumor identification [2, 3]. Bronchiolitis obliterans (BO) is a unique form of PNP-related lung lesions [5]. PNP may also damage the gastrointestinal tract [6].

PNP is serologically featured by autoantibodies to varied keratinocyte-associated proteins, commonly of IgG-type and infrequently IgA-type [2]. The autoantibodies target at desmosome-related cadherin-like molecules and plakin proteins that connect the cadherin-like molecules and cytokeratin filaments. The cadherin-like molecules represent desmoglein-1, desmoglein-3, and desmocollins-1–3. The target plakin family proteins include desmoplakin-I, desmoplakin-II, envoplakin, periplakin, plectin, plakophilin-3, and epiplakin. Autoantibodies to bullous pemphigoid antigens-1–2 and alpha 2-macroglobulin-like protein-1 are also identified [2]. Desmocollins and plakin family proteins are unique targets of PNP [2]. Particularly, antienvoplakin and antiperiplakin antibodies are diagnostic of this disorder [2]. Desmogleins-1 and -3 are also involved in pathogenesis. Antiepiplakin antibodies, positive in 72.9% in PNP [7], and antidesmoglein-3 antibodies [8] reportedly play important roles in provoking BO.

We report herein an autopsy case of PNP, associated with mantle cell lymphoma, an intractable B-cell malignancy [9]. PNP affected the oropharyngeal, respiratory and gastrointestinal mucosae. Immunosuppressive therapy accelerated systemic opportunistic infectons.

## 2. Clinical Presentation

A 70-year-old Japanese male nonsmoker visited Shimada Municipal Hospital, Shimada, Shizuoka, Japan, with complaints of dry cough, stomatitis, and sore throat lasting for 20 days. Erythematous skin lesions had repeatedly appeared for half a year. On admission, the lips and oral mucosa were painfully eroded, and hemorrhagic eruptions existed on the chest and abdomen. Periungual erythema was associated. Computed tomography showed mediastinal and para-aortic lymph node swelling and right-sided pleural effusion. Lactate dehydrogenase was elevated to 233 U/L, C-reactive protein: 7.17 mg/dL and soluble interleukin-2 receptor: 8,337 U/mL. Bone marrow aspiration demonstrated increased (47.8%) lymphocytes, expressing CD45, CD20, CD79a, CD5, and cyclin D1, and the diagnosis of mantle cell lymphoma, stage 4B, was made. No leukemic change was seen in the peripheral blood.

By indirect immunofluorescence, IgG antibodies in the 1 : 40 diluted patient serum reacted with keratinocyte cell surfaces of normal human skin sections and with transitional epithelia of rat bladder sections. Basement membrane fluorescence was negative. Immunoblot assays using human epidermal extracts demonstrated autoantibodies to envoplakin, periplakin, and bullous pemphigoid antigen-1 but did not show antibodies to desmogleins-1 and -3 and bullous pemphigoid antigen-2. Enzyme-linked immunosorbent assay (ELISA) disclosed antibodies to desmoglein-3 (index: 69.74), desmocollin-2 (optical density (OD): 0.108), and desmocollin-3 (OD: 1.068). Negative results included desmoglein-1 (index: 0.37) and desmocollin-1 (OD: 0.009). The discrepancy between the immunoblotting and ELISA for detecting desmoglein-3 was probably related to higher sensitivity of ELISA. Epiplakin was not studied.

**Figure 1:**
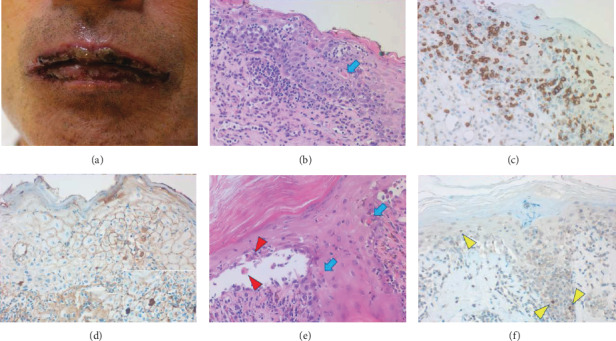
Skin biopsy of the lip and abdomen. The lips grossly reveal extensive erosion (a). Microscopically, both the spongiotic epidermis with graft-versus-host disease-like appearance and upper dermis are infiltrated by small lymphocytes (b, HE). Blue arrow indicates a Civatte body. The lymphocytes are predominantly immunoreactive for CD8 (c). After proteinase-1 digestion of the paraffin section, IgG deposition along the plasma membrane of the involved keratinocytes is proven (d). IgM is also deposited (inset). Dermal IgG positivity represents the normal distribution of IgG in tissue fluid. The abdominal skin exhibits pemphigus vulgaris-like interface blister formation. Acantholytic keratinocytes are indicated by red arrowheads, and Civatte bodies are shown by the blue arrows (e, HE). Yellow arrowheads indicate cleaved caspase-3 immunoreactivity in apoptotic keratinocytes (f).

Biopsy features of the lip and abdominal skin were consistent with PNP (Figure 1). In the lip, the regenerative epidermis revealed intercellular edema and Civatte body formation, and small lymphocytes, mainly CD8 T-cells, infiltrated both the epidermis and upper dermis. The features suggested graft-versus-host disease-like changes, showing severe interface dermatitis caused by cell-mediated immune reactions. Hyperkeratotic abdominal skin revealed blister formation along the interface (basal part of the epidermis). Both acantholytic keratinocytes and Civatte bodies were dispersed. Immunostaining using paraffin sections after prolonged protease-1 digestion [10] disclosed deposition of IgG and IgM on the plasma membrane of the involved keratinocytes. IgA was negative, serving as internal negative control. Apoptotic keratinocytes (Civatte bodies) displayed cleaved caspase-3 immunoreactivity after heat-induced epitope retrieval [11].

Modified CHOP (cyclophosphamide, hydroxydaunorubicin, oncovin, and prednisolone) therapy started, and the regimen was changed to RB (bendamustine and rituximab) therapy. The lymphoma manifested partial remission. Against PNP, steroid pulse therapy (methylprednisolone 1,000 mg/day for 3 days) started and oral methylprednisolone administration (20–125 mg/day) continued until death. The orolabial lesions were only temporarily responded to intravenous immunoglobulin infusion for five days but soon became reexacerbated to hamper eating food. During the clinical course, diagnostic radiologists suggested the possibility of BO. No gastrointestinal symptoms were recorded. No microbiological analysis was performed during the clinical course. He died of respiratory failure two months after onset.

## 3. Autopsy Findings

Residual small-sized lymphoma cells were observed in the fibrotic para-aortic (retroperitoneal) tissue. Mild lymphomatous infiltration was seen in the cholestatic liver, 150 g spleen and 100 mL ascites. Nodal involvement was unclear. The bone marrow was hypoplastic.

**Figure 2:**
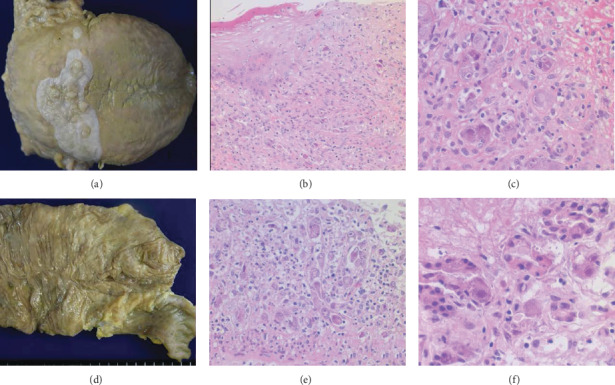
Opportunistic CMV infection in the digestive tract. The tongue is extensively eroded: the white-colored mucosa around the vallate papillae remains intact (a). Glossal erosion adjacent to intact squamous mucosa reveals infection of CMV (b, HE), and the high-powered view clarifies CMV infection in the endothelial cells (c, HE). Multifocal erosions are formed in the mucosae of the terminal ileum and cecum (d). The endothelial cells of the eroded cecal mucosa are heavily infected by CMV, and crypt epithelial cells are lost (e). CMV infection in the pancreas has provoked fat necrosis (f).

No bullous skin lesions remained, except for periungual erythema. The lips and oral/glossal and pharyngeal mucosae were severely eroded, and the erosive lesions extended to the epiglottis, trachea, and bronchi. The esophageal, gastric, small intestinal, and colonic mucosae also presented multifocal erosions. Microscopically, these eroded mucosae extensively accompanied opportunistic CMV infection. Nuclear and cytoplasmic inclusions of CMV were predominantly seen in endothelial cells, and the CMV infection resulted in severe loss of epithelial cells (Figure 2). CMV was disseminated to the adrenal gland, pancreas, liver, prostate, and kidney.

**Figure 3:**
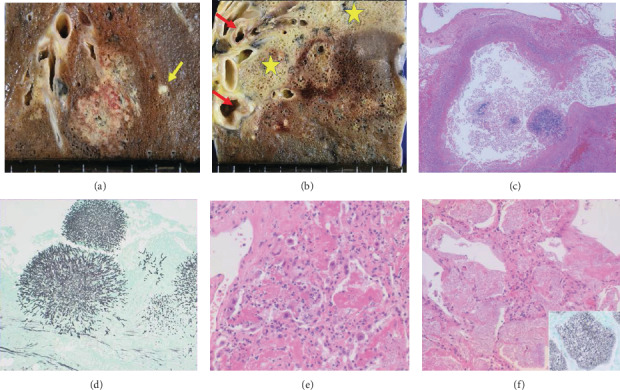
Opportunistic infections in the lung. Gross appearance of aspergillosis reveals irregular-shaped foci of infarction rimmed with hemorrhage (a and b). Mycotic embolization is indicated by the yellow arrow (a). Interstitial reactions with fibrinous exudation are seen segmentally (b: asterisks). Red arrows indicate erosions in the bronchial mucosa. Hypha-forming Aspergillus colonizes the eroded bronchiolar mucosa (c, HE and d, Grocott). CMV infection has provoked interstitial pneumonia (e, HE). Frothy intra-alveolar exudation represents pneumocystosis jirovecii (f, HE, inset: Grocott).

Both lungs showed pronounced opportunistic infections (Figure 3). Hypha-forming *Aspergillus* colonized the eroded bronchial/bronchiolar mucosa, and angioinvasive mycosis caused multifocal lung infarction, but without systemic spread. Acute interstitial pneumonia resulted from coinfection of CMV and *Pneumocystis jirovecii*. The trachea and large bronchi were coinfected by CMV and *Aspergillus*. No squamous metaplasia was noted in the tracheobronchial mucosa.

**Figure 4:**
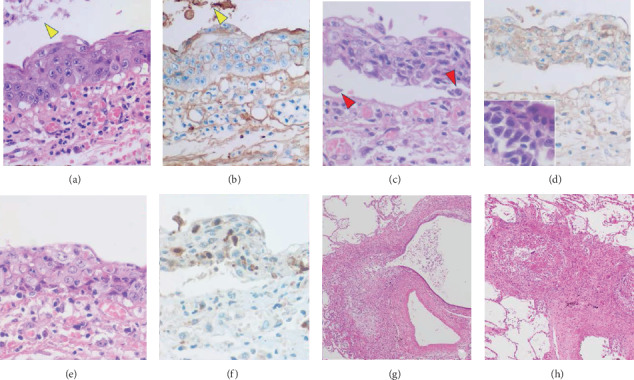
PNP-related bronchial/bronchiolar lesions. The regenerative bronchial mucosa focally with basal intercellular vesicle formation is associated with IgG deposition on the plasma membrane (a: HE, b: IgG immunostaining after proteinase-1 digestion). Intraluminal cellular debris is also labeled for IgG (yellow arrowheads). The stromal labeling represents endogenous IgG distributed in the tissue fluid. (c, HE and d, IgG) Interface blister formation with IgG deposition on the epithelial cells is demonstrated. Acantholytic cells are indicated by red arrowheads. Inset demonstrates acantholytic change of the bronchial mucosa (HE). Another part of the disorganized bronchial mucosa shows clustering of apoptotic cells immunoreactive for cleaved caspase-3 (e: HE, f: cleaved caspase-3 immunostaining). Microscopic features of BO are observed in the peripheral lung. Mucosal erosion-associated exudation has provoked secondary luminal dilatation (g, HE) and luminal obstruction (h, HE). Peribronchiolar fibrosis is noted.

PNP-related acantholytic blisters were microscopically identified in the CMV-uninfected oral floor and esophagus, as well in the bronchial/bronchiolar mucosa. Figures 4(a)–4(f) displays bronchial mucosal lesions with interface inflammation, Civatte-like apoptotic body formation, and bullous detachment. Acantholytic cells were occasionally observed. IgG deposition was identified on the plasma membrane of the bronchial epithelium in paraffin sections (frozen sections were unavailable). IgA and IgM were undetectable. Cleaved caspase-3-immunoreactive cells in the involved bronchial mucosa represented Civatte-like apoptotic bodies. Because of the absence of squamous metaplasia, the immune target was regarded to be the ciliated “nonmetaplastic” airway mucosa. Erosions in the uninfected bronchiolar mucosa induced intraluminal obstructive exudation and peribronchiolar fibrosis, typical features of BO, as illustrated in Figures 4(g) and 4(h).

**Figure 5:**
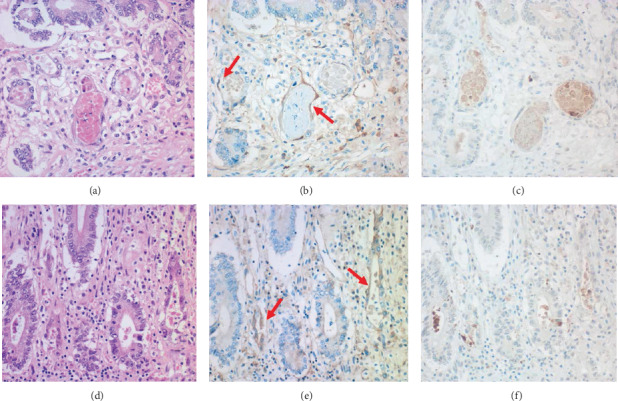
PNP-related gastrointestinal mucosal lesions: gastric antrum (a-c) and colon (d-f). Distorted pyloric glands and colonic crypts in the CMV-uninfected islands seen among extensively CMV-infected mucosae exhibit apoptotic cellular debris in the lumen (a and d: HE). Intraepithelial lymphocytes are increased in the colonic mucosa. As indicated by red arrows, IgG deposition is proven on the plasma membrane and in the cytoplasm of the involved epithelial cells (b and e: IgG immunostaining after proteinase-1 digestion). Cleaved caspase-3-immunoreactive apoptotic cells are clustered in the lumen of the diseased glands/crypts (c and f).

In the mucosae of the gastric fundus and pylorus and small and large intestine, dilated/distorted CMV-uninfected glands/crypts, seen as islands among the extensively CMV-infected mucosa, displayed intraluminal detachment of cleaved caspase-3-immunoreactive apoptotic columnar cells (Figure 5). Intraepithelial lymphocytes increased. Deposition of IgG (but not IgA and IgM) was noted on the plasma membrane and in the cytoplasm of the involved columnar cells.

## 4. Discussion

The present case of PNP manifested the mucocutaneous lesions prior to confirming mantle cell lymphoma. This happens in one-third of PNP cases [2, 3]. The patient's serum reacted not only to human epidermis but to rat urothelia commonly utilized for screening PNP [2]. The serum contained autoantibodies to varied desmosome-related proteins, including desmoglein-3, desmocollins-2 and -3, envoplakin, periplakin, and bullous pemphigoid antigen-1. Such complexed profile of autoantibodies, particularly positivity to envoplakin and periplakin, is unique in PNP [2]. Autoantibodies to desmocollins, the known targets of IgA pemphigus, are common in PNP [12]. Cytokine overproduction by lymphoma cells is hypothesized as a mechanism for producing autoantibodies in PNP. Hematologic malignancies often release cytokines, and serum interleukin-6 levels are elevated in PNP [13].

The lip and abdominal skin revealed graft-versus-host disease-like changes and pemphigus vulgaris-like interface blisters, respectively. The deposition of IgG and IgM was observed along the plasma membrane of involved keratinocytes in paraffin sections. It is known that prolonged protease digestion effectively retrieves the antigenicity of deposited immunoglobulins in formalin-fixed, paraffin-embedded sections, as sensitive as immunofluorescence using fresh-frozen sections [10]. In pemphigus vulgaris, in contrast to IgG autoantibodies, IgM autoantibodies showed no significant pathogenic roles in blister formation [14]. CD8 T-cells predominantly infiltrated the skin lesions accompanying graft-versus-host disease-like changes. CD8 T-cells function in cell-mediated pathogenesis in pemphigus vulgaris [15]. A breakdown of immune tolerance in PNP due to underlying malignancy activates autoimmunity in both humoral and cellular immunity [16, 17].

Tracheobronchial and bronchiolar mucosae multifocally revealed bullous/acantholysis-like detachment. As reported previously [5], IgG deposited on the plasma membrane of the involved epithelium accompanying accelerated apoptosis. BO in the peripheral lung was microscopically featured by bronchiolar erosion-induced intraluminal exudation and peribronchiolar fibrosis. Autoantibodies to epiplakin and desmoglein-3 reportedly cause BO in PNP [7, 8]. Hata et al. experimentally demonstrated that ectopic expression of desmoglein-3 in squamous metaplasia of the bronchial mucosa played a pathogenic role in BO [18]. In the present case, however, squamous metaplasia was scarcely seen in the bronchial mucosa, and the bullous change was observed in nonmetaplastic airway mucosa.

PNP-related gastrointestinal mucopathy was noteworthy. Miida et al. [6] reported a case of PNP with gastrointestinal involvement: multifocal erosions in colonic mucosa revealed lytic crypts with complement 3 deposition. In the present case, CMV-uninfected gastric glands and intestinal crypts, remaining as islands among extensively CMV-infected mucosa, showed luminal dilatation/derangement with apoptotic death of the columnar cells and increase of intraepithelial lymphocytes. Immunostaining on paraffin sections identified IgG deposition on the plasma membrane and in the cytoplasm of the involved epithelial cells. These findings represent PNP-related gastrointestinal involvement. The target antigen on the bronchial and intestinal epithelia remained unclear in the present case. The lack of gastrointestinal symptoms might be related to difficulty in food intake.

The direct cause of death was immunosuppression-related systemic opportunistic infections. Angioinvasive aspergillosis and interstitial pneumonia with coinfection of CMV and *Pneumocystis jirovecii* provoked lethal respiratory failure. At autopsy, the oral/glossal, gastrointestinal, and tracheobronchial mucosae were extensively infected by CMV. The virus extensively affected endothelial cells resulting in the loss of epithelial components, whereas the above-mentioned islands of PNP-related epithelial lesions occurred in the CMV-uninfected respiratory and digestive tract mucosae.

In conclusion, extensive mucosal involvement is closely related to the poor prognosis of PNP. The detailed histopathological analysis of PNP-induced mucosal lesions may contribute to better understanding of the life-threatening complications of this tumor-related autoimmune disorder. Early clinical suspicion of systemic opportunistic infection should be important for improving the patient's conditions.
